# DEMENTIA CAREGIVERS: THE IMPACT OF SOCIAL DISTANCING ON RELIGIOUS AND SPIRITUAL PRACTICE AND BEHAVIORAL SYMPTOMS

**DOI:** 10.1093/geroni/igac059.2019

**Published:** 2022-12-20

**Authors:** Katherine Britt, Kavita Radhakrishnan, Liam Fry, Kathy Richards

**Affiliations:** University of Pennsylvania, Austin, Texas, United States; University of Texas at Austin, The, Austin, Texas, United States; University of Texas at Austin, The, Austin, Texas, United States; University of Texas at Austin, The, Austin, Texas, United States

## Abstract

Prevalent among older adults with Alzheimer’s disease related dementia (ADRD), behavioral symptoms can cause adverse consequences for those with ADRD and their caregivers. Prompting earlier institutionalization and increased rapid cognitive decline, behavioral disturbances are difficult to manage. Social isolation prompted by the COVID-19 pandemic limited social and religious activities utilized by caregivers and older adults with ADRD for coping and may have affected behavioral symptoms and overall well-being. This qualitative study aimed to explore the impact of the pandemic on behavioral symptoms, religious and spiritual (R/S) utilization for coping, and well-being in caregivers and older adults with ADRD. A purposive sample of 7 home family caregivers and 7 nursing home caregivers (4 indirect family caregivers, 3 nurse practitioners) actively caring for an older adult with ADRD participated in semi-structured telephone interviews. Directed content analysis was used to analyze data. While behavioral symptoms increased overall for older adults with ADRD and social R/S practice abruptly declined, they still utilized individual R/S resources for coping such as prayers, reading religious texts, and holding a prayer book which provided a calming effect, decreased their anxiety, and prompted memories. Caregivers participated in R/S virtual groups which calmed them, connected them with others, helped them transition to sleep, and prompted mindfulness. Resources and interventions utilizing social and individual R/S for coping are warranted as they have the potential to promote cognitive stimulation, connection with others, decrease neuropsychiatric symptoms, and decrease stress.

